# Biocompatibility and acid resistance of preformed crowns in children: an in vitro study

**DOI:** 10.1007/s40368-024-00898-3

**Published:** 2024-04-25

**Authors:** T. Hogerheyde, D. Coates, L. Walsh, S. Zafar

**Affiliations:** 1 School of Dentistry, The University of Queensland, 288 Herston Road, Herston, 4006 Australia; 2 Faculty of Dentistry, University of Otago, Dunedin, New Zealand

**Keywords:** Preformed paediatric crowns, Biocompatibility, Acidity, Gingival fibroblasts, Ion release

## Abstract

**Purpose:**

To investigate the in vitro biocompatibility of human gingival fibroblasts with preformed paediatric crowns and resistance to acid exposure at levels that simulate the oral environment.

**Methods:**

This laboratory study investigated primary HGFs viability, metabolic activity, cytotoxicity, and apoptotic events on preformed metal crown discs, composite resin-coated wells, and monolithic zirconia fragments at 24, 48, and 72 h using the ApoTox-Glo Triplex assay. The PPCs were also immersed in 0.1% lactic acid, 0.2% phosphoric acid, or 10% citric acid for 7 days at 37 °C to reproduce conditions associated with dietary intake or gastric reflux. Samples were then subject to inductively coupled plasma optical emission spectrometry to quantitate the release of ions.

**Results:**

The viability of HGFs on stainless steel and CR significantly declined at 48 and 72 h, representing potential cytotoxicity (*p* < 0.05). Cytotoxicity of HGFs was also higher for stainless steel and ZR compared to control (*p* < 0.05). PMCs and ZR crowns gave minimal ion release. Meanwhile, significant quantities of metallic ions, including copper (Cu), iron (Fe), nickel (Ni), and zinc (Zn), were present in eluates from veneered-preformed metal crowns.

**Conclusion:**

As PPCs can be exposed to highly acidic environments for many years, thus the release of metallic ions from V-PMCs should form the further investigation in future studies.

## Introduction

Since the 1950s, preformed paediatric crowns (PPCs) have been widely used as one approach to treat paediatric dental caries (Innes et al. 2015). PPCs allow retention of masticatory function and preserve the dental arch until permanent successors erupt. The survivability of PPCs is paramount, whether used in the oral cavity until natural exfoliation or as a definitive long-term treatment in adulthood for conditions such as hypomineralised first permanent molars. To that end, understanding the behaviour of the oral tissues to the foreign crown materials is vital to supporting and improving their design, compositional constituents, and clinical indications.

PPCs can be manufactured from a range of materials including stainless steel, zirconia (ZR) ceramics, and composite resins (CRs) used as veneers on a base of stainless steel. The elemental atomic composition of the preformed metal crowns (PMCs) currently in use is iron (Fe; 64–70%), chromium (Cr; 18–20%), Ni (8–11%), manganese (Mn; up to 2%), and silicon (Si; up to 1%) with traces of other elements including aluminium and molybdenum (1–2%) (Zinelis et al. 2008). The Ni content of earlier versions of PMC was as high as 72%, and this was associated with Ni allergies in children (Feasby et al. 1988), which is greatly reduced in newer crowns. Some reports have suggested that the levels of Ni release from dental alloys is too low to be significant, and have argued that placing Ni-containing dental alloys is unlikely to cause allergic reactions (Bhaskar and Reddy 2010; Bica et al. 2017; Labbé et al. 2023; Gagneja 2007; Kerosuo et al. 1995; Kodaira et al. 2013; Park and Shearer 1983). However, research into whether Ni ions released from PMCs are cytotoxic and measures of cell viability, metabolic activity and apoptosis in human cells, are scarce (Yilmaz et al. 2012; Zafar and Siddiqi 2020). This is surprising given that PMCs have been shown to release metal ions (Ramazani et al. 2014). A study investigating dental hard tissue absorption of metal ions released from primary molars covered with PMCs found that levels of Fe, Ni, and Cr were 6 times higher in cementum, compared with controls (Keinan et al. 2010). Gingival fibroblast cytotoxicity from metal ion solutions released from PMCs has also been reported (Elshahawy et al. 2009). Elevated levels of Cr in hair samples of children with PMC have also been reported when compared with control groups with no PMCs; however, the levels of Cr were so low that they were deemed unlikely to cause harmful effects (Kodaira et al. 2013).

On the other hand, the aesthetic monolithic ZR crowns are composed of zirconium oxide (88–96%), yttrium oxide (4–6%), hafnium oxide (5%), an organic binder (2–5%), and a proprietary pigment (1–4%). This choice of material affects clinical outcomes of aesthetic crowns on primary maxillary teeth, with improved gingival health and low plaque levels for ZR crowns (Walia et al. 2014). In contrast, veneered-preformed metal crowns (V-PMCs) and CR strip crowns have been associated with the growth of dental plaque. However, the local effects of ion release on adjacent gingival tissues by these PPCs have not been reported. A clinical and radiographic assessment of effects of PMCs on the health of the periodontium was conducted by Sharaf and Farsi (Sharaf and Farsi 2004) and evaluated oral hygiene, gingival health, proximal contacts, crown adaptation, and crown extension. Gingivitis and interproximal bone loss were associated with poorly adapted margins or non-satisfactory placement when judged radiographically, indicating an iatrogenic effect from operator error as the primary cause, rather than effects from the material itself. As many as 42% of PMCs on primary molars may been reported to have deficient margins (Salama 1996). In addition, subgingival placement of crown margins places the crown material in contact with periodontal tissues. As the margin moves more sub-gingivally, the health of the surrounding tissues has been shown to decline (Newcomb 1974; Orkin et al. 1987; Reitemeier et al. 2002).

The oral environment is challenging for dental materials because of the range of microbial, chemical, and enzymatic actions that operate (Bhaskar and Reddy 2010; Danaei et al. 2011). The acidic milieu from diet, poor oral hygiene, or systemic conditions such as diabetes or gastric reflux is a key concern. Crowns have similarities to stainless steel orthodontic appliances where metal corrosion issues been explored, and which share similar metal composition to stainless steel (House et al. 2008; Kerosuo et al. 1995). Release of nickel (Ni) ions from Ni-containing stainless steel orthodontic wires has been reported (Bhaskar and Reddy 2010; Dwivedi et al. 2015). This has helped fuel the development and manufacturing of improved materials for orthodontic appliances, with higher corrosion resistance and lower cytotoxicity for human cells (Zhang et al. 2014). As Ni is a common cause of allergic reactions, and allergies to Ni occur more frequently than those to other metals, (Ramazani et al. 2014) affecting approximately 10% of the general population (Danaei et al. 2011). Clinical symptoms associated with the Ni allergy include allergic dermatitis, asthma, and mucosal ulcers (Burrows 1986; Hildebrand et al. 1989). Moreover, Ni ions can cause damage to the periodontium, which may act as a modifying factor for periodontal disease (Gursoy et al. 2007; Naranjo et al. 2006; Pazzini et al. 2009). While most patients may experience minimal or no side effects, individuals with predisposing allergies or medical conditions could be adversely affected. Given the above considerations, the present study had two main objectives: first, to assess the biological responses of HGF cells grown on paediatric crown materials, and second, to measure the release of ions from PPCs immersed in acidic solutions.

## Materials and methods

The study sample and preparation of crown materials*:* The study sample consisted of three types of commercially available PCs: NuSmile ZR Zirconia™ (ZR; NuSmile, Houston, Texas, United States), NuSmile Signature™ (NS; NuSmile, Houston, Texas, United States), and 3 M ESPE Stainless steel crowns (PMC; 3 M ESPE, St Paul, Minnesota, United States), with n = 3 samples per test condition.

Stainless steel crowns discs were created from type 316 L stainless steel sheets (3 M) using a metal punch (PPS-7 Power Punch Set, Metalmaster; TW) to give 3.18 mm diameter samples. Meanwhile, ZR fragments (~ 30–40 mg) from ZR crowns (NuSmile^®^) were simultaneously tested. Finally, TPH Spectra CR low viscosity resin (Dentsply Sirona Inc.; Charlotte, USA) was used to coat 96-well plates using a ball burnisher. Materials were disinfected using 70% ethanol for 20 min then rinsed 3 times in 0.15 M phosphate buffered saline (PBS) immediately prior to use.

Establishment of primary HGF cultures*:* Gingival tissue was collected from two healthy patients aged 15–17 years following gingivectomy procedures undertaken as part of orthodontic treatment. Human Research Ethics Committee (HREC) approval was obtained from the Ethics Committee of Royal Bruisbane Hospital and The University of Queensland (Ethics Approval No. HREC/2019/QRBW/57321). Two HGF cultures were established using Zafar et al. 2014 methodology from cell suspensions that were maintained in a growth medium of Dulbecco’s minimal essential medium with GlutaMAX (DMEM with GlutaMAX; Gibco^®^ Invitrogen, MA, USA) containing antibiotic–antimycotic reagent (0.5 µg/mL; Invitrogen), gentamycin (0.25 µg/mL; Invitrogen) and supplemented with 10% foetal bovine serum (FBS, Gibco® ThermoFisher Scientific; MA, USA) (Zafar et al. 2014). HGFs were used between passages 3 and 8.

Cell viability, cytotoxicity, and apoptosis (ApoTox-Glo Triplex) assay*:* The biocompatibility of HGFs cultured on crown materials (PMC discs, Zr fragments or control) was measured using the ApoTox-Glo Triplex assay (Promega; Madison, USA). This assay assesses cellular viability, cytotoxicity, and apoptosis events. HGF cells were seeded at 1.5 × 10^4^ cells/cm^2^ into 96-well plates in 100 µL growth media, then incubated for 24, 48, and 72 h. For each time point, the plates were processed according to the manufacturer’s instructions. Viability (400_Ex_/505_Em_)/cytotoxicity 485_Ex_/520_Em_ absorbance was measured using an Infinite 200 Pro Tecan spectrophotometer (Tecan Group Ltd; Männedorf, CH), whereas apoptosis luminescence was read on a Tecan Spark multimode plate reader (Tecan Group Ltd; Männedorf, CH). This assay was performed in triplicate (*n* = 3) for each HGFs culture.

*Acid exposure assays of preformed crowns:* Whole PPCs were immersed in 2 mL of 0.1% lactic acid, 10% citric acid, or 0.2% phosphoric acid solutions in 24-well plates for 7 days at 37 °C. The materials used were PMCs, V-PMCs, and ZR crowns (*n* = 3 crowns samples per condition). Liquid samples were then analysed for elements of interest using a PerkinElmer Optima 8300 (PerkinElmer; Waltham, USA) dual-view inductively coupled plasma optical emission spectrometer (ICP-OES) located at the Queensland University at Central Analytical Research Facility (CARF).

Briefly, the sampling system consists of a peristaltic pump set at 0.44 L/min for analysis, an ESI SC Fast Prep (version 2.9) auto dilutor and sampler with an S400V syringe unit, cross-flow nebuliser with an argon gas flow rate of 0.60 L/min. Plasma was generated by a solid-state 40 MHz radio frequency generator power set at 1500 Watts. The plasma argon flow rate was set at 12 L/min, and the argon gas for the auxiliary was set at 0.8 L/min. The ICP-OES dual monochromators for ultraviolet and visible range emission had a wavelength range of *λ* = 165 nm to *λ* = 800 nm. Emitted wavelengths were measured using a sealed charged coupled device (CCD) detector, which was cooled to a temperature between -7 and -8 °C with an integrated Peltier cooler. Multi-elements calibration standards and a 5 µg/mL Lu and 2.5 µg/mL Y internal standard solutions were prepared gravimetrically that cover the concentration range of all samples. All elemental readings of ≤ 2 ppm were excluded from further analysis.

Statistical analysis: The data were tabulated on a Microsoft Excel spreadsheet (Version 16.30, Microsoft Corp.) and then imported into GraphPad PRISM 9.0 software (GraphPad Software, San Diego, CA, USA) for statistical analysis and creation of appropriate graphs. Specific data analysis tests were performed for biocompatibility and acid immersion experimental data using a two-way ANOVA. The levels of statistical significance of *P* ≤ 0.05 was considered statistically significant.

## Results

**Fig. 1:**
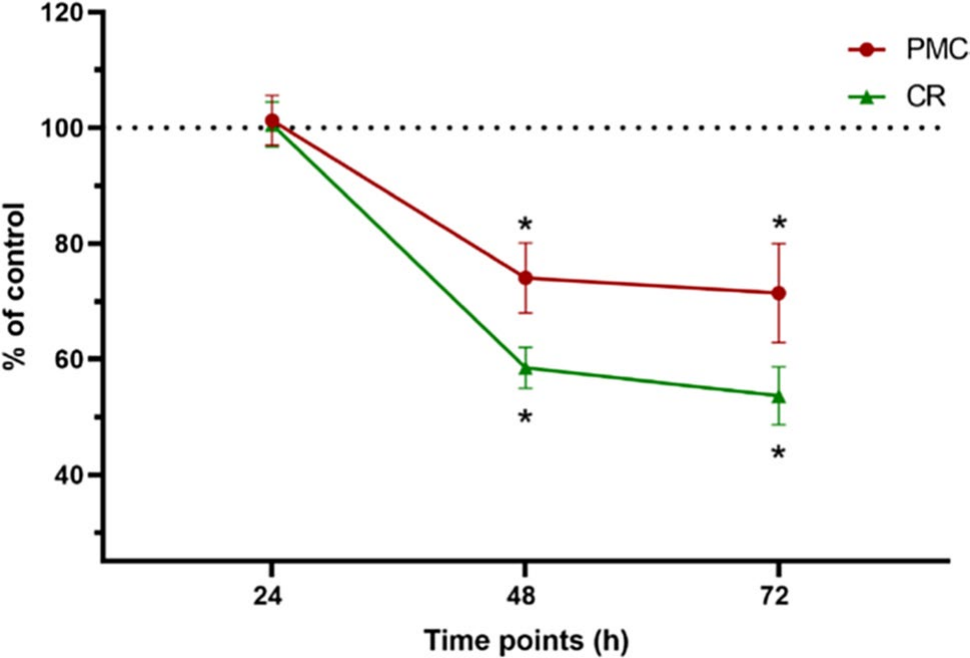
*MTT* mitochondrial dehydrogenase activity of human gingival fibroblasts grown on *PMC* preformed metal crown discs or composite resin-coated wells (CR; 2) as a percentage of the tissue culture plastic (TCP; dash line represents percentage of control at 100%) control at 24, 48, and 72 h (*n*=3; technical replicates, HGF-2). Asterisks indicate significant changes (*p* < 0.05) compared with TCP. Mean ± SEM

Effects of crown materials on HGF viability*:* The ApoTox-Glo assay was used to measure the viability of HGFs grown on PMC and ZR. In both HGF-1 and HGFs-2 cultures, viability on PMC and ZR materials was higher for all time points compared to control (Fig. 1).

**Fig. 2:**
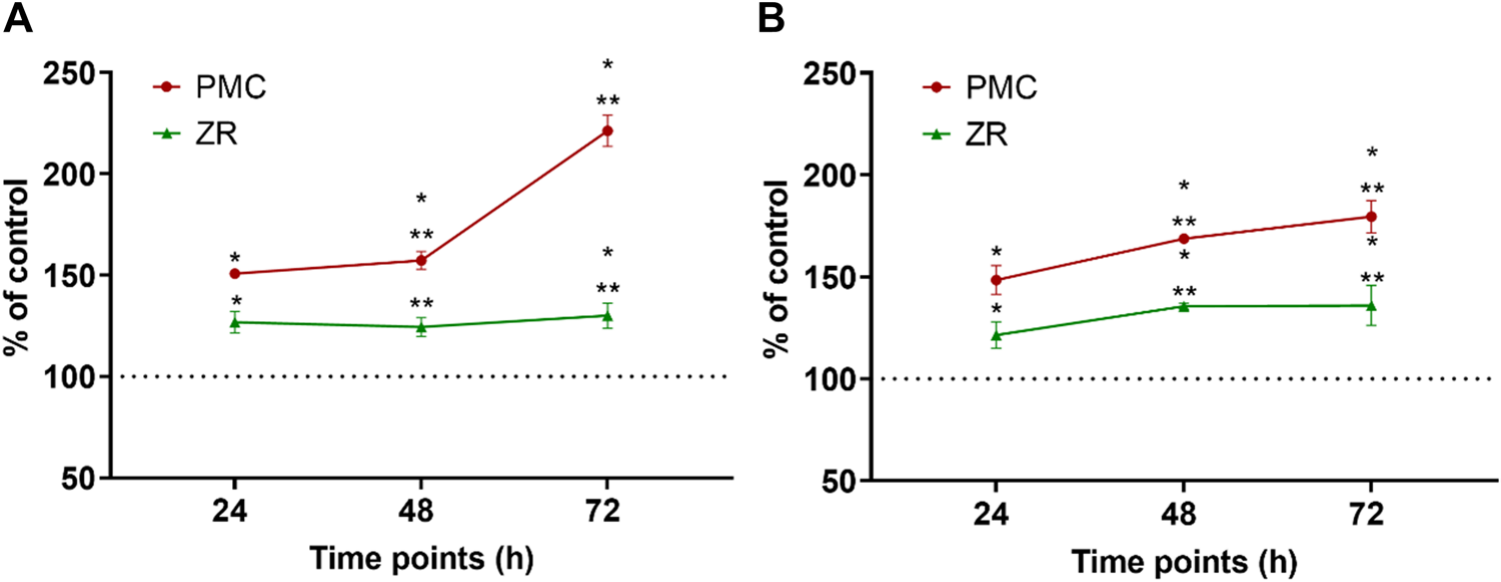
Cell viability of human gingival fibroblasts (HGFs) grown on PMC discs and ZR fragments compared with control at 24, 48, and 72 h (*n*=3; technical replicates). **a** Cytotoxicity assay with HGF-1 cells established from donor 1.** b** Viability assay conducted with HGF-2 cells established from donor 2. Dotted line represents percentage of control at 100%. Asterisks indicate significant changes (p < 0.05) compared with the untreated control (*) or competing crown material (**). Mean ± SEM

Effects of crown materials on HGF cytotoxicity*:* HGFs from both donors showed consistently higher cytotoxicity to PMC and ZR materials, compared to control (Fig. 2A, B). For cells from HGF-1, cytotoxicity values were significantly (*p* ≤ 0.05) elevated for PMC at 48 h and ZR at 24 and 72 h. Meanwhile, for cells from HGF-2, cytotoxicity values for both materials at each time points were above those for control. Neither material was associated with significantly higher cytotoxicity than the other.

**Fig. 3:**
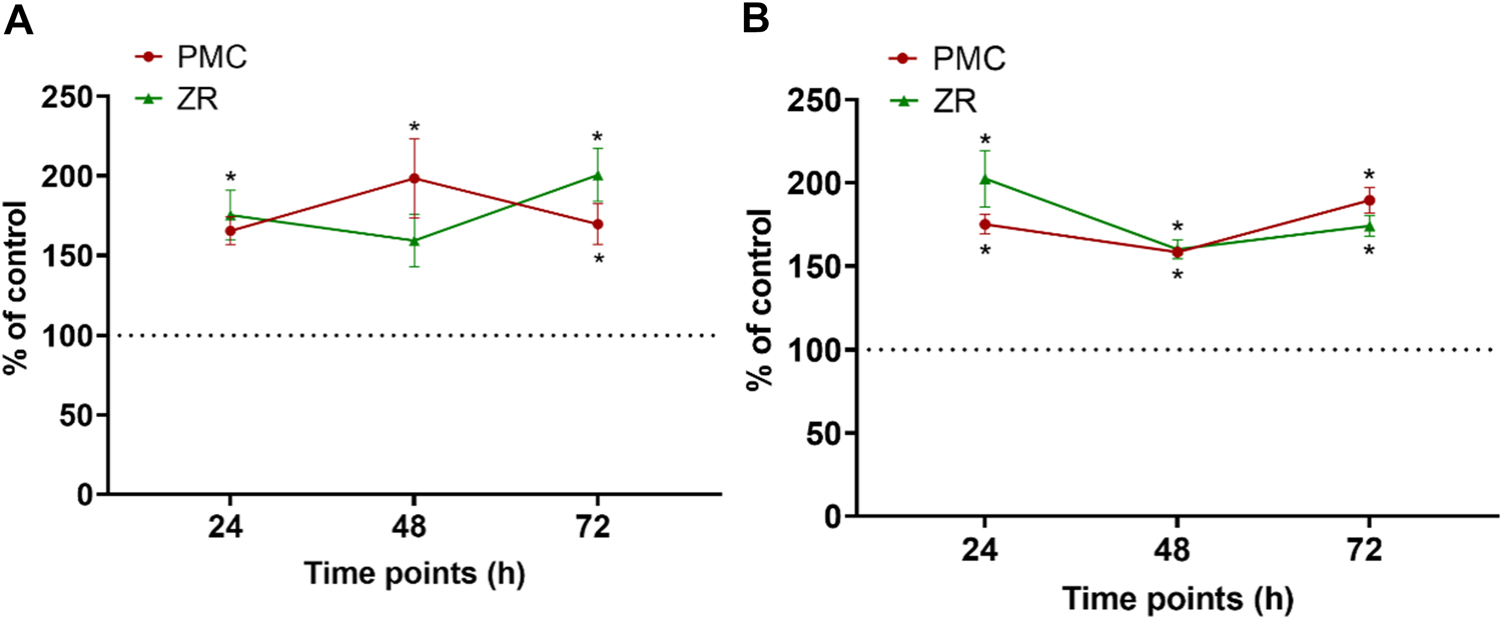
Cytotoxicity of PMC discs and ZR fragments compared with control on human gingival fibroblasts (HGFs) at 24, 48, and 72 h. **a** Cytotoxicity assay with HGFs established from HGF-1. **b** Cytotoxicity assay conducted with HGFs-2 established from HGF-2. Dash line represents percentage of control at 100%. Asterisks indicate significant changes (p < 0.05) compared with the untreated control. Mean ± SEM

Effects of crown materials on HGF apoptosis*:* The presence of caspase 3/7 apoptotic markers in HGFs seeded onto crown materials was recorded using the ApoTox-Glo assay. For HGF-1, apoptotic signalling increased with extended exposure to PMC discs being significantly higher at 72 h (Fig. 3A). Meanwhile, cells from HGF-2 were significantly less responsive, with marginal increases in apoptotic events over time (Fig. 3B). The apoptotic profile for cells from both donors seeded onto ZR showed increasing rates of apoptosis after 2 or 3 days. After this time, apoptosis declined steadily for cells from HGF-1, while the rate of apoptosis for cells from HGF-2 increased at 72 h.

**Fig. 4:**
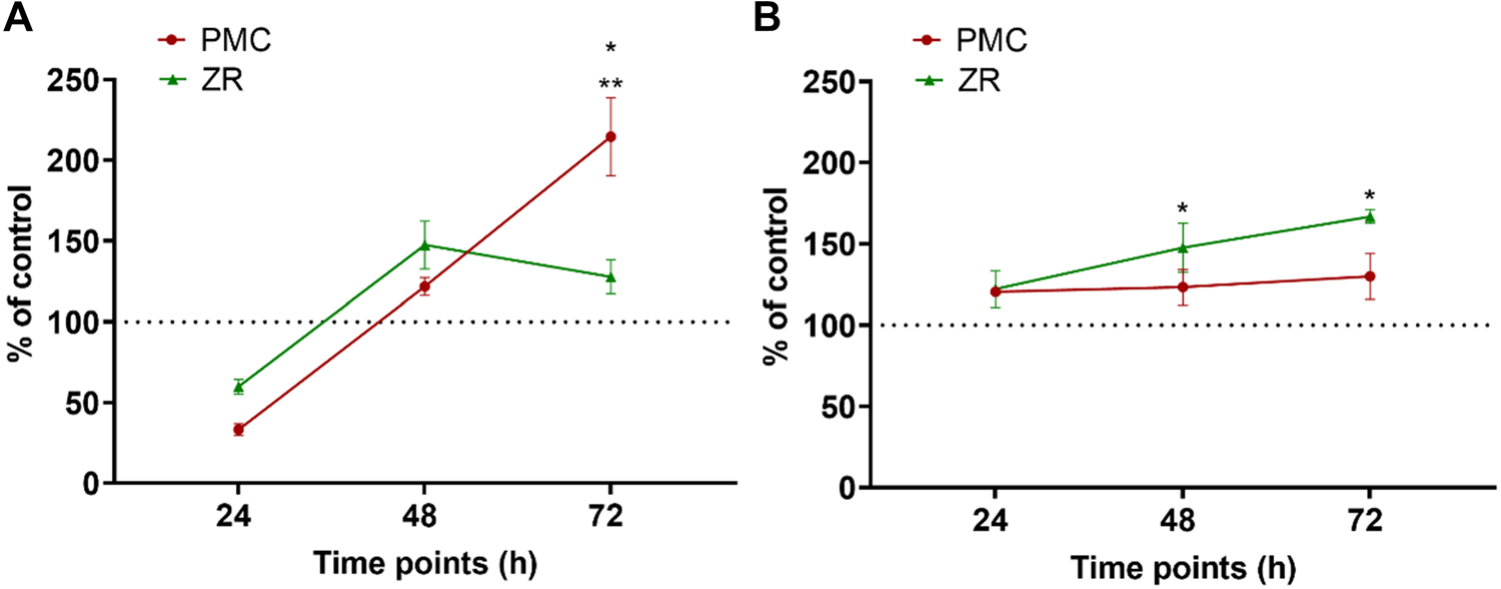
Caspase 3/7 levels in human gingival fibroblasts (HGFs) grown on PMC discs and ZR fragments compared with control at 24, 48, and 72 h. **a** Apoptosis assay conducted with HGFs-1 established from HGF-1 **b.** Apoptosis assay conducted with HGFs-2 established from HGF-2. Dash line represents percentage of control at 100%. Asterisks indicate significant changes (*p* < 0.05) compared with the untreated control (*) or competing crown material (**). Mean ± SEM

Release of ions from acid-exposed paediatric crowns*:* PPCs were immersed in acidic solutions for 7 days at 37 °C to determine their solubility by measuring ion release using ICP-OES. The minimum threshold for inclusion was ≥ 2 ppm. V-PMC was the most reactive, with high levels of Cu, Fe, Ni, and Zn ions **(**Fig. 4**)** in the acidic elutes. The most destructive solution was 0.2% phosphoric acid followed by 10% citric acid, and lastly 0.1% lactic acid. PMCs released marginal quantities of Fe and Si ions. ZR crowns were the least reactive material, with minimal acid solubility.

## Discussion

The results of the present in vitro study show that certain paediatric restorative materials could be to some degree cytotoxic to human gingival fibroblasts depending on the associated factors and the oral environment. Paediatric preformed crowns are essential for preserving the paediatric dentition, and can be manufactured from various materials (Alrashdi et al. 2022; Midani et al. 2019). The severity of early childhood caries means a range of materials may be needed to satisfy a child’s functional and aesthetic needs. Preformed paediatric crowns remain in contact with periodontal tissues until natural exfoliation and as such, these materials need to perform without local or systemic impacts on the host (Randall 2002). This includes the capacity of crown materials to withstand environmental stresses including oral acidity. In rare instances, some dental materials can participate in immunological reactions (Hosoki et al. 2015). For example, previous preformed metal crown iterations which contained significantly more nickel than contemporary counterparts (Feasby et al. 1988). The in vitro biocompatibility of periodontal cells grown on preformed paediatric crown materials has not previously been documented. Specifically, the ability of human gingival fibroblasts to withstand short-term exposure to preformed paediatric crowns has, until now, not been explored, even though the response of gingival fibroblasts to composite resins or ceramic materials has been reported (Gautam et al. 2012, 2016; Pabst et al. 2014). Cellular responses to materials such as monolithic zirconia are less well understood (Alrashdi et al. 2022; Yanover et al. 2021). As a result, this study chose to assess the biocompatibility of zirconia ceramics using a series of assays.

The results of the viability assay showed increase in cell viability for both preformed metal crown and zirconia crowns compared to control. Whether similar metabolic trends occur for preformed metal crown or zirconia crowns in vivo remains unknown. There is a need to conduct similar studies using in vivo animal models. Similar trends were observed in apoptosis assay. This observation could be a result of an increase in cell density, rather than the number of cells per se. The results of the study showed no real indication of a large induction of apoptosis. As described by others, the early release of ions may explain also explain the findings (Elshahawy 2011; Milleding et al. 2003; Rizo-Gorrita et al. 2019).

Human gingival fibroblasts from both donors showed consistently higher cytotoxicity to preformed metal crown and zirconia materials, compared to control. The exposure of periodontal cells to cytotoxic resin-based materials has been extensively reported, with unreacted monomers having potentially detrimental effects on periodontal tissues (Al-Hiyasat et al. 2005; Ausiello et al. 2013; Darmani et al. 2007). Both host and environmental factors may contribute to monomer release. For instance, daily exposure to dietary acids can adversely affect the composite resin matrix-filler interface, increasing the potential for loss of material (Tanthanuch et al. 2014). Similarly, the corrosion of stainless steel crowns can release ions which then produce cytotoxic cellular effects (Elshahawy et al. 2009). Stainless steel orthodontic bracket eluates after immersion in acidic solutions have been shown to induce genotoxic effects on human gingival fibroblasts, due to the release of ions (Loyola-Rodríguez et al. 2020). In addition, free radicals may damage cellular DNA and lead to mutagenic or carcinogenic outcomes (Faccioni et al. 2003; Zinelis et al. 2004). In the present study, no significant difference in cytotoxicity was found between cells cultured on preformed metal crown discs or on zirconia fragments (Fig. 3A, B; *p* > 0.05). However, human gingival fibroblasts experienced noticeably higher cytotoxicity when grown on crown materials compared with the control (*p* < 0.05). A study investigating the biocompatibility of human gingival fibroblasts cultured on ceramic-based materials found cytotoxicity levels significantly decreased over time (Rizo-Gorrita et al. 2019). Those authors postulated that the release of ions from ceramics as responsible for the initial cytotoxic response. However, in the present study, cells were more viable when grown on stainless steel discs after 48 h, when compared with zirconia. This is despite the fact that zirconia ceramics are considered highly biocompatible (Gautam et al. 2016; Yanover et al. 2021).

Overall, zirconia ceramics show a low corrosive potential with minimal cytotoxic constituents that would minimally affect cellular behaviour. Even so, the effects of increasing oral acidity on ion release is underreported. The present study found zirconia crowns immersed in ultrapure H_2_O were largely unaffected. However, the addition of acidic solutions initiated the release of aluminium and silicon ions, albeit in small quantities. These elements are common ingredients in ceramic-based materials such as lithium disilicate or zirconia-reinforced lithium disilicate, with reported low cytotoxicity (Elshahawy 2011; Milleding et al. 2003). Others have described chemical interactions between gastric acid solutions and monolithic zirconia that result in smoother surfaces, which may indicate that a corrosive process has occurred (Kukiattrakoon et al. 2010; Milleding et al. 2002; Sulaiman et al. 2015). Some ion leaching due to corrosion of zirconia could have occurred in the solutions used in the present study.

**Fig. 5:**
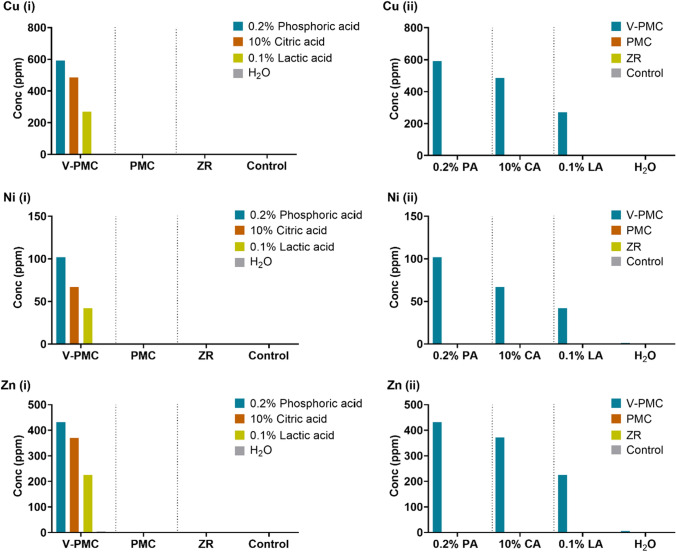
PPC solubility. Cu, Ni, and Zn ion release categorised by (**i**) materials and (**ii**) acidic solutions

The leaching of ions from veneered-preformed metal crowns into acidic solutions raises potential health concerns (Kasprzak et al. 2010). In particular, the release of the metallic ions copper, iron, nickel, and zinc warrants further investigation. The concentration of zinc in veneered-preformed metal crown eluates was 5 ppm (H_2_O), 225 ppm (0.1% lactic acid), 371 ppm (10% citric acid), and 432 ppm (0.2% phosphoric acid) (Fig. 5). Others have shown that mouse fibroblasts exposed to Ni and Cu metal salt solutions displayed in vitro cytotoxicity at 10 ppm (Milheiro et al. 2016). In contrast, the release of copper and nickel ions from veneered-preformed metal crowns in 0.2% phosphoric acid was 592 ppm and 102 ppm, respectively. A similar article testing the acid resistance of glass ionomer cement restorative materials reported 0.1% lactic acid to be the least destructive solution (Perera et al. 2020). The present study also observed that veneered-preformed metal crowns released significantly less material when immersed in 0.1% lactic acid, compared with the 10% citric acid and 0.2% phosphoric acid solutions.

Elsewhere, others have found that zinc ions released from dental materials could impair cellular viability. Similarly, the leaching of nickel ions has the potential for eliciting delayed hypersensitivity reactions, as well as carcinogenic and mutagenic cellular events (Zafar and Siddiqi 2020). Overall, metallic ions released from veneered-preformed metal crowns have been found to be highly cytotoxic (Elshahawy et al. 2009). Interestingly, this contrasts with traditional preformed metal crowns which are largely unaffected.

A limiting factor of this study was the sole focus on HGFs. It would be valuable for future researchers to consider the response of gingival epithelial cells or immune cells to the tested materials. In addition, expanding the number of primary donors to validate suspected trends would be highly beneficial.

## Conclusions

The results of the present study show that paediatric restorative materials could be slightly cytotoxic to human gingival fibroblasts depending on the associated factors and the oral environment. Whether cytotoxicity is limited to the early phases of growth or continues over a longer duration remains to be determined. The release of metallic ions from veneered-preformed metal crowns should form the basis of future studies. It would be prudent to expose human gingival fibroblasts to similar ion concentrations (measured by molality) to better understand the relativity of these cytotoxic effects.

## Data Availability

The data will be available on request.
